# Effects of a reduced efficacy of the KCC2 co-transporter in temporal lobe epilepsy: single neuron and network study

**DOI:** 10.1186/1471-2202-16-S1-P5

**Published:** 2015-12-18

**Authors:** Anatoly Buchin, Gilles Huberfeld, Richard Miles, Anton Chizhov, Boris Gutkin

**Affiliations:** 1 École normale supérieure, Laboratoire des Neurosciences Cognitives, Group for Neural Theory, Paris, France; 2 Peter the Great St.-Petersburg Polytechnic University, St.-Petersburg, Russia; 3 Neurophysiology Department, Pitie-Salpetriere Hospital, UPMC, Paris, France; 4 Epilepsie de l'Enfant et Plasticité Cérébrale, INSERM U1129, Paris, France; 5 Institut du Cerveau et de la Moelle Epiniere, Cortex et Epilepsie Group, Paris, France; 6 Ioffe Physical Technical Institute, Computational Physics Laboratory, St.-Petersburg, Russia; 7 Higher School of Economics, Moscow, Russia

## 

**Figure 1:**
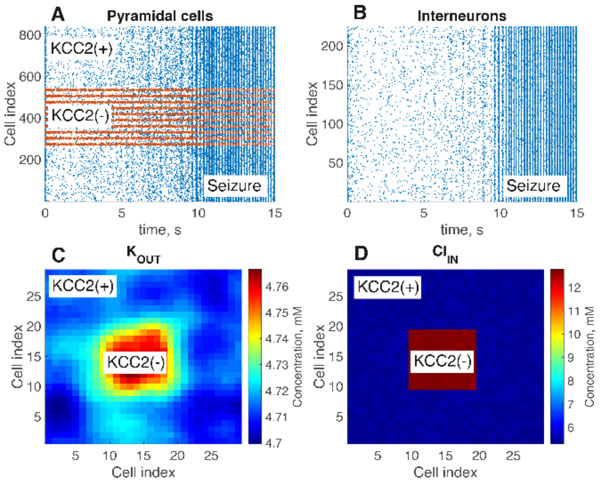
**A raster plot of pyramidal cell population firing**; B raster plot of interneuron firing; C spatial distribution of extracellular potassium; D intracellular chloride distribution.

Epilepsy is one of the most common neurological disorders. Seizures in about 40% of patients with temporal lobe epilepsies are pharmaco-resistant [1]. In surgically removed hippocampal tissue from these patients, the KCC2 cotransporter is absent or non-functional in about 20 % of subicular pyramidal cells [2]. KCC2 normally assures the maintenance of low intra-neuronal chloride levels [3] and also regulates potassium levels [4]. Chloride concentration changes in the remaining pyramidal cells due to intensive GABAergic input during seizures could reverse the effects of GABA currents from inhibitory to excitatory [5,6]. Such changes may shift a pyramidal cell into a periodic bursting regime associated with ictal discharges. Using a detailed biophysical model of a single cell incorporating these mechanisms of ionic homeostasis and a neural network model, we show that decreasing the activity of KCC2 pump leads to repetitive seizure-like firing in the pathologic network due to increased extracellular potassium and intracellular chloride (Fig. 1). This model provides insights into how a dysregulation of pyramidal cell chloride homeostasis due to reduced levels of the KCC2 cotransporter may lead to seizures in the epileptic human subiculum.
